# Rare Case of a 20p13 Duplication Trisomy With Craniostenosis

**DOI:** 10.7759/cureus.61949

**Published:** 2024-06-08

**Authors:** Radu Eugen Rizea, Ligia Gabriela Tataranu, Amira Kamel, Alexandru Vladimir Ciurea, Karina Lidia Gheorghita

**Affiliations:** 1 Neurosurgery, Emergency Clinical Hospital Bagdasar-Arseni, Bucharest, ROU; 2 Neurosurgery Department, Carol Davila University of Medicine and Pharmacy, Bucharest, ROU; 3 Neurology, Pediatric Neurology Private Practice, Bucharest, ROU

**Keywords:** autism spectrum disorder, type 1 diabetes, craniosynostosis, epilepsy, duplication 20p syndrome, trisomy 20

## Abstract

Duplication 20p or partial trisomy 20 is a rare chromosomal anomaly characterized by duplication of the short arm of chromosome 20, with various clinical abnormalities. Despite complete trisomy 20, which usually leads to prenatal death, partial trisomy 20 can manifest with variable phenotypes, from mild to severe manifestations. Here, we present a rare case of an 8-year-old boy diagnosed with trisomy 20, epilepsy with focal seizures of genetic origin, craniosynostosis, type 1 diabetes, and autism spectrum disorder. Duplication 20p is a complex diagnostic and presents a therapeutic challenge due to its diverse clinical manifestations. To succeed in the intricacy of such a unique and challenging case, a comprehensive clinical and genetic assessment must be performed.

## Introduction

Full trisomy 20 is an exceptionally rare entity that frequently leads to embryonic or early fetal loss. Given its rarity and dismal prognosis, the exact prevalence and pathophysiological impact of full trisomy 20 are revealed by case reports and retrospective studies. Chromosomal duplications are linked to genetic diversity and the pathogenesis of various diseases. This syndrome's particularity consists of rarity and a wide range of clinical manifestations. The symptoms of partial trisomy can range from mild to severe, depending on the characteristics of the duplicated segment and the variable genes involved [1-3]. The duplication of the short arm of chromosome 20, known as duplication 20p, means the presence of additional genetic material on this chromosomal region. The size of this duplication can be very different among individuals, including a lot of important genes for neurocognitive developmental processes. The specific clinical manifestations associated with duplication 20p are linked to the particular duplicated segments on chromosome 20. As these unique variations can significantly impact the clinical manifestations, the results will vary from mild phenotypic presentations to severe ones. The intricacy and variability of the clinical features highlight the major importance of an elaborate genetic analysis, not only to enhance the comprehension of underlying mechanisms but also to provide an accurate diagnosis, prognosis, and tailored management strategy for each individual [4]. Patients with chromosome 20p duplication often display a broad spectrum of clinical characteristics, such as global developmental delay, intellectual disability, speech and language impairment, specific facial features, and clinodactyly. Most of the individuals affected by duplication 20p reveal instantly noticeable, specific facial features. These characteristics may be facial abnormalities such as hypertelorism or a wide nasal bridge, aiding in the clinical diagnosis of the syndrome. The possible manifestations include different types of skeletal anomalies such as clinodactyly or scoliosis, heart defects that can range from minor atrial septal defects to severe congenital heart diseases, and neuropsychiatric issues. The severity of symptoms is generally correlated with the size of the duplication [4-6]. Usually, karyotyping, aCGH (Array Comparative Genomic Hybridization), and FISH (fluorescence in situ hybridization) tests are used to diagnose trisomy 20p. These techniques determine the exact boundaries of the duplication, which is essential for predictions and treatment strategies [3,4]. The treatment strategies for duplication 20p are primarily supportive and symptomatic, adapted to every single patient. Standard treatment programs are represented by occupational and speech therapies. The treatment team includes geneticists, pediatricians, neurosurgeons, orthopedic surgeons, and neurologists. Genetic counseling is necessary for affected families to discuss the condition's heritability and future recurrence risk [6,7].

## Case presentation

**Figure 1:**
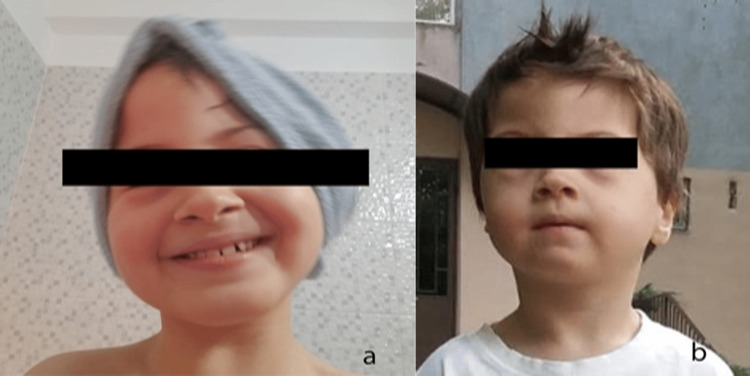
Clinical aspects (a) and (b) Clinical aspects - the child presents a specific type of scaphocephaly: sphenocephaly, with a "wedge-shaped" head.

We present the case of an eight-year-old boy diagnosed with partial trisomy 20p and multiple comorbidities (Figure 1a-1b). The child was diagnosed at the age of four years old, and no significant prenatal history was recorded in his medical history. The patient was delivered at 36 weeks of gestation by C-section, with the fetus in breech presentation, weighing 2500 g, a cranial circumference of 31 centimeters, a pinpoint anterior fontanelle, and scaphocephaly. He exhibited severely delayed neuropsychomotor development at different age stages, febrile seizures, focal motor seizures, both when awake and asleep, feeding difficulties, behavioral disorders, delayed expressive and receptive language development, sleep disturbances, marked psychomotor agitation, attention deficit, stereotypies, and moderate intellectual disability.

**Figure 2:**
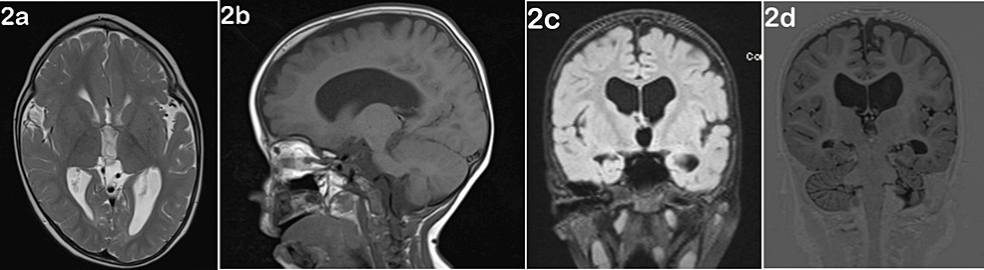
Cerebral MRIs Cerebral MRIs indicate the pathognomonic aspect of "boat-shaped" skull, with a narrow and long cranial shape and a metopic ridge: (a) axial T2; (b) sagital T1; (c) coronal FLAIR; (d) coronal T1 SPACE.

A brain MRI (magnetic resonance imaging) performed at one year of age revealed sphenocephaly, widened pericerebral spaces, sharp gyri, cortical relief with decreased deep and subcortical white brain matter, and deepening of supratentorial pericerebral spaces (Figure 2). At the age of three months, the neurosurgical team recommended corrective surgery for scaphocephaly, which was refused by the family.

Until the age of four years, the child was only treated for developmental disorders, undergoing physical therapy and language stimulation therapy. Due to the low number of febrile seizures, no anticonvulsant treatment was prescribed. Focal motor epileptic seizures appeared around the age of four years and were successfully treated with carbamazepine in the correct dose adapted for age and weight. In addition, around the age of four years, the child developed type 1 diabetes, with initial symptoms of polydipsia, polyphagia, and polyuria. These symptoms resolved after receiving a combination therapy of fast-acting and long-acting insulin, which remained stable over time. Considering the complexity and uniqueness of this case, it was decided to perform a whole genomic sequencing test, which revealed three genetic mutations classified as VUS (a variant of uncertain significance): COL4A3BP, ARID1B, and DEPDC5, and a gene classified as pathogenic suggestive for partial trisomy 20: heterozygous duplication seq GRCh37 dup (20) (p13p12.1). At the age of eight, the patient presents with moderate intellectual disability, severe receptive and expressive language delay, and behavior disorders suggestive of autism spectrum disorder. Under treatment, the patient maintains a balanced metabolism and does not have epileptic seizures.

## Discussion

The association of partial trisomy 20p with scaphocephaly and type I diabetes diagnosed at pediatric age suggests the uniqueness of these associated comorbidities. The progressive onset of neurological symptoms (the patient exhibited consistently delayed neuropsychomotor development from birth until the present moment), initial diagnosis of scaphocephaly, clinical onset of type 1 diabetes symptoms at the age of four years, and polymorphic epilepsy seizures led to the suspicion of genetic pathology. Following genetic testing, the suspicion of a genetic syndrome identified as partial duplication 20p was confirmed. The lack of specific symptomatology for partial trisomy 20p, namely cardiac, digestive, and skeletal disorders of joints or scoliosis, led to the delay in the correct diagnosis of the case. We can assume that the skeletal changes in this genetic syndrome can also be associated with changes in the neurocranium, such as craniostenosis in this case. The genetic cause of the onset of a metabolic pathology at pediatric age, specifically type I diabetes, is reconfirmed.

Chaabouni et al. reported a case of trisomy 20p associated with a small distal 20q, which originated from de novo duplication. The authors considered that this paternal origin duplication could be explained by either of two mechanisms. The first supposed mechanism is related to a translocation between the chromosomes 20 homologous that will sustain the development of two derivates, while the second mechanism refers to an event that occurs next to the premeiotic stage, described as an unbalanced crossover within the inversion segment of chromosome 20. The results of the study concluded that it was an example of an almost pure 20p duplication, which differentiates from other case reports by down-slanting palpebral fissures, short philtra length, and hexadactyly [8].

Another report of an intricate case was published by Bartolini et al., describing a child with a confirmed 17.98 Mb duplication of chromosome 20p associated with a 422.3 kb duplication on chromosome 3p that was maternally inherited. This was one of the rarest cases with de novo partial trisomy 20p. Moreover, neuroimaging modifications were described, as a temporal arachnoid cyst and a pineal gland cyst were observed. However, the authors concluded that these findings may be common in pediatric patients, and it is not sure whether they were correlated to trisomy 20p or not, and they suggest that maternal treatment with lamotrigine for epilepsy could be an influential factor regarding the issue [9].

Similar cases were described by Kennedy et al. in 2024. As in our case, both the cases presented by these authors are partial trisomies 20p with the association of other various defects with the addition of genetic material [10].

Considering the complex aspect of our case, we suggest early genetic testing from birth in cases of craniocerebral malformations. We believe that the particular aspect of the presented case is due to the association of duplication with the other three VUS genes. The existence of these severe cases could be avoided by genetic testing of pregnant mothers as a standard method in their evaluation during pregnancy. We also recommend a multidisciplinary approach to these cases, considering their complexity.

## Conclusions

Based on the aforementioned statements, we consider that the association of 20p partial trisomy with scaphocephaly and type 1 diabetes in the pediatric age confers uniqueness and complexity to this particular case. The genetic basis of metabolic disorders, especially juvenile diabetes, emphasizes the significance of early genetic screening in cases of association with cerebral and cranial malformations. The distinctive aspects of this case highlight the importance of prenatal genetic testing in order to prevent the occurrence of these severe cases through their early identification and management and to avoid affecting the quality of life of both these patients and their families. Due to the complex nature of such cases, a multidisciplinary team approach is recommended to obtain maximum benefit and provide appropriate care.
